# The Efficacy and Safety of Dr. SKS Hair Booster Serum (a Cocktail of Micronutrients and Multivitamins) in Adult Males and Females With Androgenetic Alopecia: An Open-Label, Non-randomized, Prospective Study

**DOI:** 10.7759/cureus.37424

**Published:** 2023-04-11

**Authors:** Stuti Khare

**Affiliations:** 1 Department of Dermatology, Elements of Aesthetics, Mumbai, IND

**Keywords:** micronutrients, minerals, vitamins, pattern baldness, alopecia, androgen

## Abstract

Background

Androgenetic alopecia (AGA) is frequently encountered in dermatological practice; however, there is a lack of approved treatment. At present, only three therapies have been approved for on-label use in androgenetic alopecia: minoxidil, finasteride, and lower-level laser therapy. Micronutrients are primary elements in the normal hair follicle cycle, and their role in androgenetic alopecia is a growing matter of research nowadays. This study aims to investigate the clinical efficacy and safety of Dr. SKS Hair Booster Serum, a cocktail of micronutrients and multivitamins (copper, niacinamide, hyaluronic acid, thiamine, riboflavin, and biotin), in male and female patients with androgenetic alopecia.

Methods

We did an open-label, non-randomized, multicenter, prospective study across five hair clinic chains in India (Mumbai, Hyderabad, Jabalpur, Balaghat, and Nagpur). Eligible participants were patients with a confirmed diagnosis of androgenetic alopecia based on clinical examination and trichoscopic findings, age of 18 years or older, and any gender. Each patient received Dr. SKS Hair Booster Serum, 1 ml in volume, once a month by mesotherapy or derma roller/derma pen for up to six months. All patients were subjected to a 60-second hair count test (comb test), hair pull test, global photographic assessment (GPA), trichoscopy assessment, patient self-assessment questionnaire, and safety assessment at baseline and six months after the treatment.

Results

One thousand patients (500 males and females each) with androgenetic alopecia were analyzed. There was a significant reduction in hair fall with bulb (<0.0001) and without bulb (<0.0001) six months after the treatment versus baseline. There was a significant improvement in the number of hairs removed per pull (<0.0001), global photographic assessment score (<0.0001), hair growth rate (<0.0001), follicular hair density (<0.0001), vellus hair density (<0.0001), and terminal hair density (<0.0001) six months after the treatment versus baseline. The majority of patients (95%) were satisfied with six-month treatment of Dr. SKS Hair Booster Serum. No major adverse events were reported during the study.

Conclusion

Dr. SKS Hair Booster Serum was found to be a safe and effective treatment for androgenetic alopecia, with 95% patient self-assessment score.

## Introduction

Androgenetic alopecia (AGA) (referred to as “pattern hair loss”) is the most prevalent cause of hair loss worldwide. It mainly affects the frontal and parietal regions of the scalp in up to 70% of males and 40% of females and has a significant detrimental psychological and emotional impact on afflicted individuals [1]. It is a genetically predetermined disorder caused by an excessive response to androgen, which is characterized by gradual miniaturizations of terminal hair follicles through a progressively shorter anagen phase and the eventual transition of hair cycle on the scalp from terminal to intermediate, to vellus hair in a distinctive pattern. Androgenetic alopecia is categorized into two types: 1) male-pattern hair loss or male-pattern baldness and 2) female-pattern hair loss or female-pattern baldness. Male-pattern hair loss typically shows the recession of the vertex and frontotemporal hairline to develop a characteristic M shape and often leads to partial or complete baldness. On the contrary, female-pattern hair loss manifests as diffuse apical hair loss, affecting the wider anterior part of the hair and exhibiting no frontal hairline recession, and rarely progresses to total baldness [2].

To date, only three treatments, topical minoxidil, topical finasteride, and lower-level laser therapy, have been approved by the US Food and Drug Administration (USFDA) for androgenetic alopecia. All these treatments have been associated with several adverse events. As such, pruritus, scalp irritation, irritant and allergic contact dermatitis, and facial hypertrichosis are observed with minoxidil [3-5]. Finasteride (1 mg) is mainly used in dermatological practice and has been found to be associated with erectile dysfunction and decreased libido [6], hepatic functional disorder, unilateral mammary hypertrophy and palpitations, febricula, and headache [6-7]. Moreover, low-level laser therapy is linked to acne, mild paresthesia such as burning sensation, dry skin, headache, pruritus, the temporary onset of telogen effluvium, and the presence of dysplastic or malignant lesions on the scalp [8]. Other drug treatments such as progesterone, zinc salts, azelaic acid, flutamide, spironolactone, and dutasteride and surgical modalities such as scalp microneedling, platelet-rich plasma, and hair transplantation are frequently used in clinical practice, without showing promising leads with encouraging results [4]. Therefore, exploring for more precise therapies is required for androgenetic alopecia.

There are approximately 100,000 hair follicles on the human scalp [1,9]. In the absence of alopecia, 90% of these hair follicles are in the anagen phase and need vital elements including proteins, vitamins, and minerals to develop healthy hairs efficiently [10]. Deficiencies in vitamins and trace minerals may result in hair loss. Micronutrients are crucial elements in the normal hair follicle cycle and play an important role in cellular turnover, a frequent occurrence in the matrix cells in the follicle bulb that are rapidly dividing.

More recently, micronutrients and multivitamins have garnered much attention for the treatment of androgenetic alopecia. Dr. SKS Hair Booster Serum, a novel formulation consisting of 10 mg/L copper, 5 mg/L niacinamide, 0.25 mg/L hyaluronic acid, 0.2 mg/L thiamine, 0.2 mg/L riboflavin, 0.025 mg/L biotin, and water (aqua) as required, has been developed to promote hair growth and has received FDA approval (AP/RANGMANTH/SC/2022/56459/7SEP2022/1) and provisional patent approval from the Indian Patent Office. The present study sought to investigate the clinical efficacy and safety of Dr. SKS Hair Booster Serum, a cocktail of micronutrients and multivitamins, in male and female patients with androgenetic alopecia.

## Materials and methods

Methods

We did an open-label, non-randomized, multicenter, prospective study across five hair clinic chains (Mumbai, Hyderabad, Jabalpur, Balaghat, and Nagpur) in India from June 2021 to August 2022. Regardless of gender, patients with a confirmed diagnosis of androgenetic alopecia and age of 18 years or older were included. Androgenetic alopecia was diagnosed based on clinical examination and trichoscopy findings (i.e., hair shaft thickness heterogeneity, brown/white peripilar sign, yellow dots, pinpoint white points, focal atrichia, and scalp pigmentation). The main exclusion criteria were abrasion or abnormalities to the scalp, a recent history of local infections of the head, allergic reactions, active seborrheic dermatitis, or thyroid gland disease. The study was approved by the local Institutional Ethics Committee (ISDEC/NR-3/CM/2023) and conducted in accordance with the principles stated in the Declaration of Helsinki, and the subjects provided written consent for their participation in the study.

The Preparation of the Formulation

The formulation is bottled into glass vials of 5 ml each. The formulation is stable and can be stored at room temperature (below 25°C). The final composition of Dr. SKS Hair Booster Serum is demonstrated in Table 1.

**Table 1:** Final composition of Dr. SKS Hair Booster Serum

Number	Constituents	Quantity
1	Copper	10 mg/L
2	Niacinamide	5 mg/L
3	Hyaluronic acid	0.25 mg/L
4	Thiamine	0.2 mg/L
5	Riboflavin	0.2 mg/L
6	Biotin	0.025 mg/L
7	Water (aqua)	As required

Injections of the Formulation to the Scalp

In each patient, 1 ml of Dr. SKS Hair Booster Serum was injected into the superficial layer (the dermis) of the scalp by tiny infusion through insulin syringe, mesotherapy, or through derma roller/derma pen in a monthly session, with four-week interval between two sessions. The treatment is continued up to six months (equivalent to six sessions). The initial changes and visible results were observed from the second to the third month after the treatment. All efficacy and safety outcomes were assessed at baseline and six months after the treatment.

Patients were advised to follow few instructions after each treatment session: 1) Avoid combing immediately after session, and keep at least one-hour gap. 2) Avoid wearing helmet for one hour after session so that the serum can effectively absorb into the scalp. 3) Do not touch the scalp with your hands for at least one hour after session. 4) Do not wash your hair immediately after session, and keep a minimum of two-hour gap. 5) Do not apply other formulations on the scalp earlier than three hours. 6) Avoid sauna or pool for one day.

Evaluation parameter

Severity Assessment

The severity of androgenetic alopecia was assessed using the Hamilton-Norwood scale [11] comprising types I-VII. The severity was categorized into three types: mild (I and II), moderate (IIA, III, IIIA, and IV), and severe (IVA, V, VA, VI, and VII) [12].

Subjective Assessment

Sixty-second hair count test (comb test): Patients were instructed to comb their hair for 60 seconds starting at the vertex and combing forward, prior to shampooing in the morning. The hair was combed over a towel or pillowcase of contrasting color so that any shed hairs could be adequately visualized. Subjects then collected the shed hairs. The number of hairs collected was counted and divided as hair fall with bulb and hair fall without bulb at baseline and six months after the treatment.

Hair pull test: A bundle of approximately 50-60 hairs was grasped between the thumb, index finger, and middle finger and pulled from the base close to the scalp. The hair was firmly tugged away from the scalp. The extracted hairs were counted at baseline and six months after the treatment, and values were reported as the mean number of hairs per pull.

Global photographic assessment (GPA): Standardized clinical photographs of the head were captured for clinical assessment at baseline and six months after the treatment. The vertex and superior frontal areas of the scalp were photographed using a standardized technique. Photographs were assessed by two independent dermatologists who scored each image from 0 to 10, where 0 denotes no growth and 10 denotes full, thick hair growth. The scores were averaged and compared between baseline and six months after the treatment.

Trichoscopic assessment: The parameters measured using TrichoScan® Professional version 3.7.27.124 (Tricholog GmbH and Datinf GmbH, Freiburg, Germany) were hair growth (defined as the length of hair grown per two days), follicular hair density (defined as number of hairs per cm^2^), percentage of anagen hair, percentage of telogen hair, and density of terminal and vellus hair (defined as the number of hairs per cm^2^).

The participants were contacted to shave the hairs from the investigational site two days prior to each assessment visit. The investigational site was chosen in such a way that hair in the close vicinity can be combed for shaving two fingers away from the parting or on the receding hairline of the frontotemporal regions or on the vertex. The mask was applied, and hairs were pulled through it and shaved, leaving a neat space of 1 cm^2^. Then, dye was applied to the masked surface area and cleaned after 15 minutes with alcoholic solution. Photograph was captured using a camera, positioning the lens by pressing into the wet assessment area such that no bubbles were trapped. The recorded images were loaded into TrichoScan software, and analysis proceeded automatically. The same area should be identified and examined at baseline and six months after the treatment.

Patient self-assessment: A prevalidated hair growth questionnaire was given to all the patients consisting of four questions, where 0 denotes extremely dissatisfied and 10 denotes extremely satisfied with the efficacy of the investigational product at the end of the treatment (Table 2).

**Table 2:** Patient self-assessment hair growth questionnaire

Number	Question	Possible response (on a scale of 0-10)
1	Growth of hair	Extremely dissatisfied > extremely satisfied
2	Amount of noticeable new hair	Extremely dissatisfied > extremely satisfied
3	Visibility of the scalp	Extremely dissatisfied > extremely satisfied
4	Rate of hair loss	Extremely dissatisfied > extremely satisfied

Safety assessment: Adverse experiences were observed throughout the study period.

Outcomes

The primary efficacy outcomes were as follows: 1) mean change in hair fall with bulb, 2) mean change in hair fall without bulb, 3) mean change in hairs per pull, 4) mean change in hair growth, 5) mean change in hair density, 6) mean change in percentage of anagen hair, 7) mean change in percentage of telogen hair, 8) mean change in vellus hair density, 9) mean change in terminal hair density, and 10) patient self-assessment scores for hair growth. The safety endpoints were the incidence of major adverse events.

Statistical analysis

Continuous data are reported as mean and standard deviation, while categorical data were presented as frequency and percentage. All efficacy parameters were compared from baseline to six months after the treatment using a paired t-test. A p-value of <0.05 was considered statistically significant. All statistical analysis was performed using Statistical Package for Social Sciences (SPSS) software version 22 (IBM SPSS Statistics, Armonk, NY).

## Results

One thousand patients with androgenetic alopecia were analyzed to assess the efficacy and safety of Dr. SKS Hair Booster Serum. The mean age was 37.43 ± 9.89 years (range: 22-46), and half of the patients (50%) were male. With respect to severity, 25.7% of the patients had mild, 37.5% had moderate, and 36.8% had a severe type of androgenetic alopecia. Table 3 outlines the baseline demographic characteristics of the study population.

**Table 3:** Baseline demographic characteristics AGA: androgenetic alopecia

Characteristics	Percentage of patients
Total patients	1,000
Mean age, y	37.43 ± 9.89 (22-46)
Male	50%
Family history of AGA	37.2%
History of other medical diseases	23.4%
Mean duration of hair fall, y	3.0 ± 1.34 (1-5)
History of past treatment of AGA	27.5%
Severity of hair fall	
Mild	25.7%
Moderate	37.5%
Severe	36.8%

Sixty-second hair count test (comb test)

**Figure 1:**
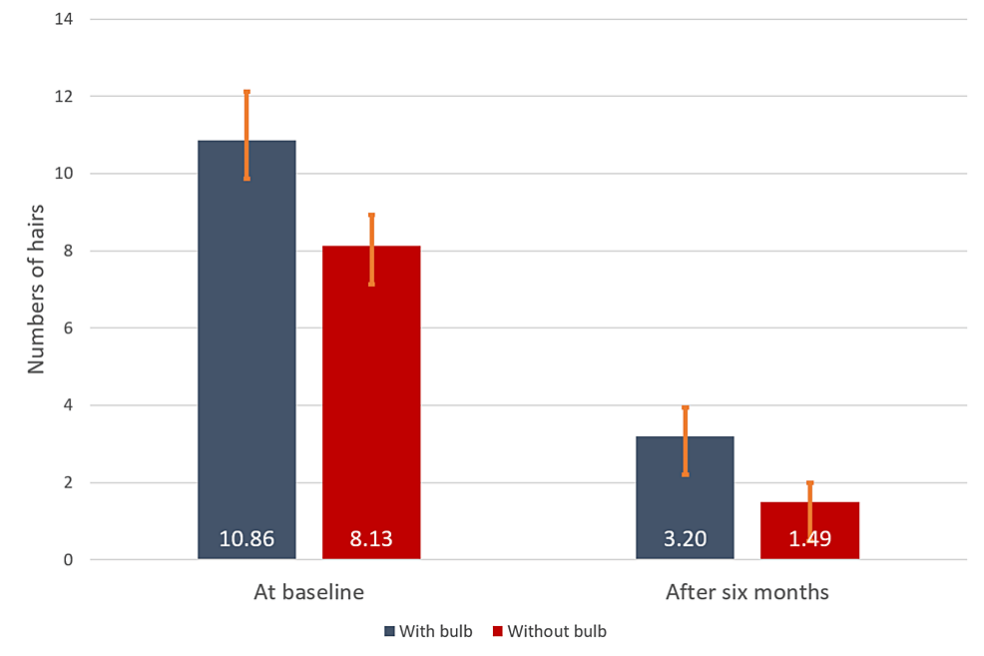
Number of hair fall with bulb and without bulb at baseline and six months after the treatment

As depicted in Figure 1, there was a significant reduction in hair fall from 10.86 ± 1.27 to 3.20 ± 0.81 (70.5%) (p < 0.0001) with bulb and 8.13 ± 0.74 to 1.49 ± 0.50 (81.7%) (p < 0.0001) without bulb at six months after the treatment versus baseline.

Hair pull test

**Figure 2:**
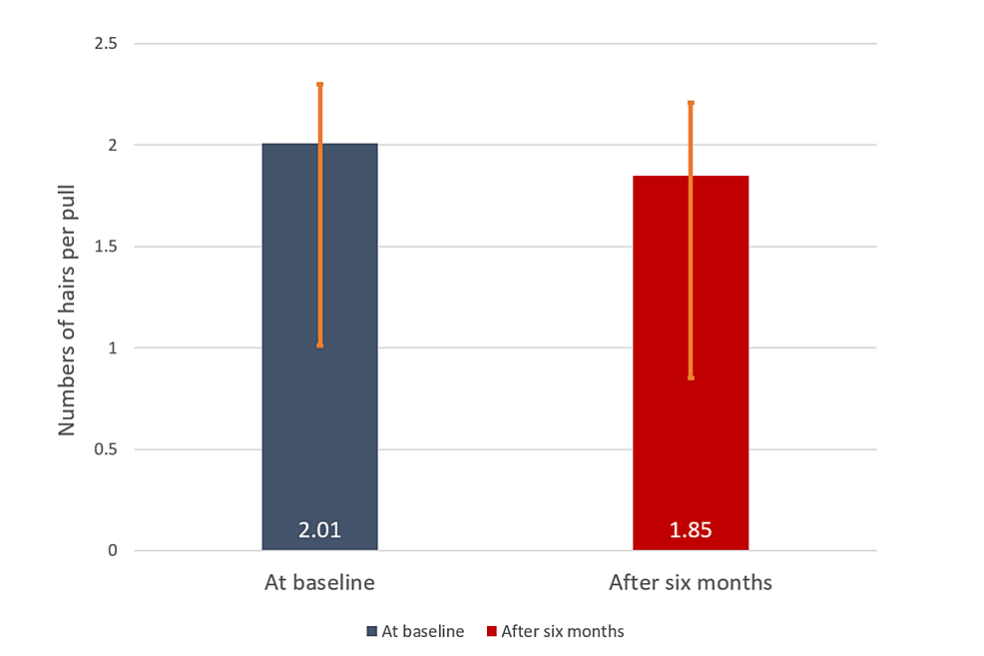
Number of hairs removed per pull

The mean number of hairs removed per pull was significantly (p < 0.0001) reduced from 2.01 ± 0.29 to 1.85 ± 0.36 (8.0%) six months after the treatment versus baseline (Figure 2).

Global photographic assessment score

**Figure 3:**
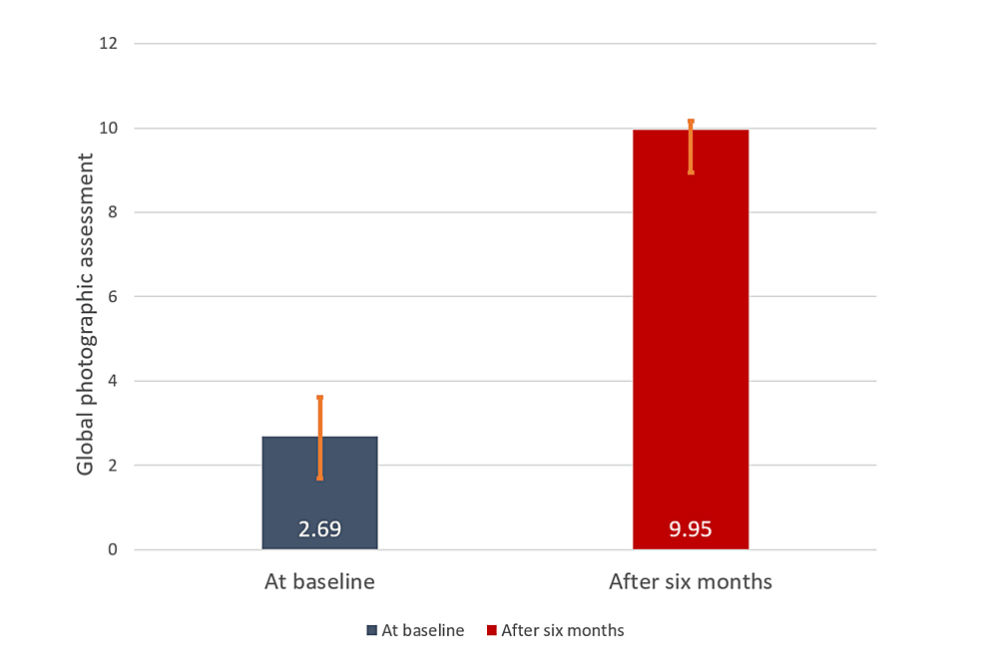
Global photographic assessment score at baseline and six months after the treatment

The mean global photographic assessment (GPA) score was improved from 2.69 ± 0.93 to 9.95 ± 0.21 (p < 0.0001) at six months after the treatment versus baseline, resulting in 73.0% change indicative of satisfactory hair growth (Figure 3).

**Figure 4:**
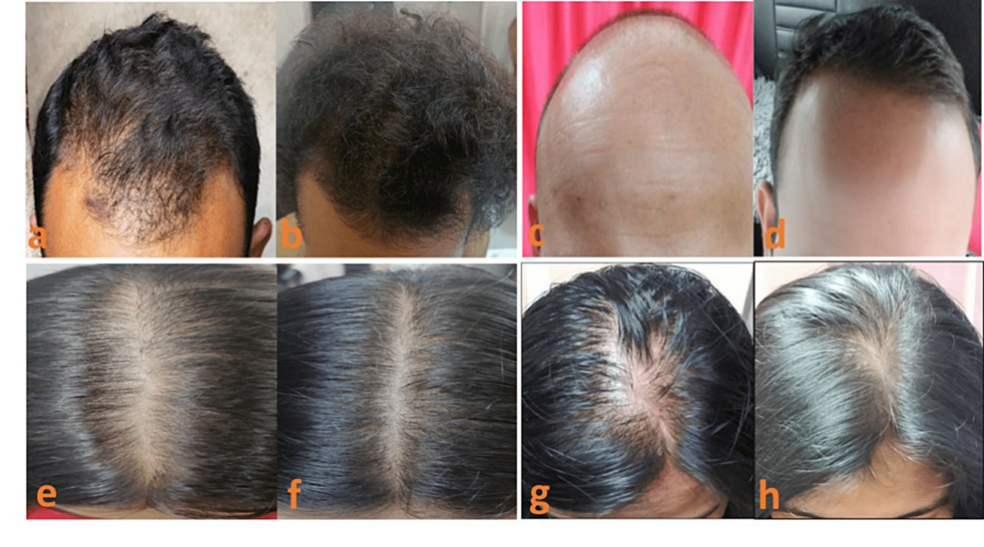
Significant hair growth in male-pattern (a, b, c, and d) and female-pattern (e, f, g, and h) androgenetic alopecia six months after the treatment versus baseline

This improvement was evident in photographs captured at baseline and six months after the treatment (Figure 4).

Trichoscopic assessment

There was a percentage improvement of 52.0% and 18.3% in hair growth and follicular hair density. The mean percentage of anagen and telogen remained similar throughout the study period. There was a percentage improvement of 18.2 and 4.6 in vellus and terminal hair density, respectively, six months after the treatment versus baseline (Table 4).

**Table 4:** Results of trichoscopy assessment

Parameters	At baseline	After six months	Paired difference	95% confidence interval		P-value
				Lower	Upper	
Hair growth (µm) (length of hair grown/two days)	253.25 ± 0.85	527.68 ± 13.21	-274.43	-275.21	-273.65	<0.0001
Follicular hair density/cm^2^	299.13 ± 1.72	354.48 ± 3.34	-19.91	-20.08	-19.76	<0.0001
Percentage of telogen hair	14.55 ± 1.12	14.55 ± 1.12	-0.00	-0.004	0.000	0.157
Percentage of anagen hair	83.42 ± 1.39	83.41 ± 1.39	0.00	-0.000	0.006	0.083
Vellus hair density/cm^2^	41.73 ± 0.49	49.31 ± 0.87	-7.50	-7.66	-7.52	<0.0001
Terminal hair density/cm^2^	129.24 ± 0.86	135.63 ± 0.48	-6.39	-6.45	-6.32	<0.0001

Patient self-assessment

After receiving treatment up to six months, 95% of the patients were completely satisfied, and only 5% of the patients were dissatisfied. The mean hair growth questionnaire (comprising four questions) score for patients satisfied with the treatment was 40 ± 0.21, which is considerably higher than the values reported for patients dissatisfied with the treatment (16.2 ± 0.93).

Safety assessment

No skin intolerance was reported during the study. No adverse event was reported throughout the treatment.

## Discussion

The present study examined the clinical efficacy and safety of Dr. SKS Hair Booster Serum in male and female patients with AGA. The main findings of the present study were as follows: 1) There was a significant reduction in hair fall with bulb, hair fall without bulb, and the number of hairs removed per pull at six months versus baseline. 2) There was a significant improvement in GPA score, as well as hair growth, follicular hair density, vellus hair density, and terminal hair density assessed by trichoscopy. 3) The percentage anagen and the percentage of telogen remained similar throughout the study period. 4) Ninety-five percent of the patients were satisfied with the six-month treatment of Dr. SKS Hair Booster Serum.

The alterations in hair cycle dynamics, specifically shorter anagen phase and prolonged telogen phase, has been considered to be the main pathophysiological factor in developing androgenetic alopecia. In patients with androgenetic alopecia, the excessive activation of the androgen receptor causes follicular shrinkage through a gradually shorter anagen phase, leading to thinner and shorter hair follicles that may not even eventually pass through the epidermis in the end. On the other hand, the duration of the telogen phase is either constant or prolonged. As a result of this, the anagen-to-telogen ratio is reduced from 12:1 to 5:1. The hair follicle miniaturization during the shorter anagen phase and constant or prolonged telogen phase results in the conversion of terminal hairs into secondary vellus hairs. In patients with androgenetic alopecia, the terminal-to-vellus hair ratio is reduced from >6:1 to <4:1 [1,13].

Dr. SKS Hair Booster Serum enriched with micronutrients and multivitamins is a completely noninvasive, non-surgical, easygoing, easily accessible, and highly effective medical therapy that facilitates hair regeneration and regrowth. The main constituents of this serum are copper [14,15], niacinamide [16,17], hyaluronic acid [18,19], thiamine [20], riboflavin [21,22], and biotin [23-25], all of which have been proved to be effective as the sole therapy for the treatment of androgenetic alopecia.

The present study found 52.0% and 18.3% improvement in hair growth and follicular hair density with six-month treatment of Dr. SKS Hair Booster Serum, as evidenced by TrichoScan®. These values were numerically comparable with that reported by Majeed et al. (hair growth: 17.36%; follicular hair density: 7.97%, at three months versus baseline) [13]. Moreover, we found a significant reduction in hair fall and no change in the percentage of anagen and the percentage of telogen. The participants experienced significant improvement in hair texture and hair volume as measured by a self-assessment questionnaire at the end of the treatment as compared to baseline. Marzani et al. reported that a lotion containing sandalwood odorant was effective in reducing hair fall and promoting hair growth with an increase in hair density, hair shaft diameter, percentage of anagen hair, and mass hair index [26]. Another study published by Majeed et al. asserted that hair serum formulation containing freeze-dried coconut water, amla extract, selenopeptide, sandalwood odorant, peanut shell extract, and tetrahydropiperine as key constituents has significantly improved hair growth rate, hair density, vellus hair, and terminal density after six months of treatment versus baseline; however, no significant change in the anagen-to-telogen ratio was reported [13].

Dr. SKS Hair Booster Serum works to strengthen, restore, and promote hair growth. It is used to treat a variety of hair conditions: 1) male-pattern baldness; 2) female-pattern baldness; 3) telogen effluvium due to stress, coronavirus disease (COVID-19), weight loss, hair loss, etc.; 4) anagen effluvium in cancer patients; and 5) alopecia areata and alopecia universalis. Dr. SKS Hair Booster Serum has successfully treated more than 30,000 patients non-surgically globally. With this study, we firstly put forward the scientific evidence of our product.

The main limitation of the study is that the hair serum product was not compared with currently available treatment modalities for androgenetic alopecia. Further randomized study with an active comparator and long-term follow-up is recommended to gather more supportive evidence.

## Conclusions

Dr. SKS Hair Booster Serum is a highly effective and safe treatment for androgenetic alopecia, with 95% patient self-assessment score. It accelerates hair growth, increases hair density, and reduces hair fall in both males and females with androgenetic alopecia. It is a well-tolerated treatment alternative for androgenetic alopecia with favorable cosmetic outcomes.
